# Winners of the analytical science advances poster awards at the 16th international symposium on hyphenated techniques in chromatography and separation technology

**DOI:** 10.1002/ansa.202000085

**Published:** 2020-07-29

**Authors:** Ali Moussa, Haibin Li, Zhanyao Hou, Paul Trevorrow, Sebastiaan Eeltink

**Affiliations:** ^1^ Department of Chemical Engineering Vrije Universiteit Brussel (VUB) Brussels Belgium; ^2^ Department for Pharmaceutical and Pharmacological Sciences University of Leuven (KU Leuven) Leuven Belgium; ^3^ Department of Organic and Macromolecular chemistry Ghent University Ghent Belgium; ^4^ Executive Journals Editor, Wiley Chichester UK

From 29 till 31 January 2020, the 16th International Symposium on Hyphenated Techniques in Chromatography and Separation Technology (HTC‐16) took place in Ghent, Belgium. The HTC conference series is one of the premier platforms for state‐of‐the‐art developments in separation technologies and hyphenated techniques that is organized every 2 years under the auspices of the Royal Flemish Chemical Society and the Royal Society of Chemistry.

The HTC‐16 conference, chaired by Sebastiaan Eeltink and Frederic Lynen, was attended by over 260 participants working in industry and academia from 26 different countries. Moreover, the conference was celebrating its 30th anniversary. The congress was preceded by 1‐day short‐courses on multidimensional liquid chromatography (LC) and gaschromatography‐mass spectrometry (GC‐MS), and cannabis analysis. Over the course of the following 3 days, 132 oral presentations were presented covering fundamental and practical aspects of liquid phase and gas chromatography, including automated sample preparation, UHPLC‐MS, 2D‐ and 3D‐LC, GC × GC, and supercritical fluid chromatography (SFC) coupled to mass spectrometry, data mining, and curation. Furthermore, emerging applications were discussed, which included biopharmaceutical analysis, proteomics, lipidomics and metabolomics, and profiling of food products among other topics. Some of the conference highlights included:

- The presentation of the HTC Innovation Award lecture by LC‐GC to Professor Ryan T. Kelly from the Brigham Young University (USA) covering innovative work on 1D and 2D separations for in‐depth single‐cell and nanoscale proteomics.
- Many excellent talks of young emerging scientists. HTC is traditionally geared toward young scientists. A dedicated parallel session was in place providing opportunities for young investigators to present oral presentations in 15‐min slots or to highlight their main research results during 5‐min poster‐flash presentations.
- The HTC tube, which was a new initiative providing participants the possibility to present a 3‐min video to communicate their research results in a creative manner. Completely different approaches were followed making it a fun and interesting session.
- A lecture on the advantages and limitations of HILIC in the second dimension of comprehensive two‐dimensional liquid chromatographic separations presented by Professor De Villiers (Stellenbosch University, South Africa). His contribution was selected by the scientific committee and industry board as the most innovative lecture of the conference, for which he received the HTC‐Award.
- The plenary lecture presented by Professor Pat Sandra, one of the Gurus of separation science, who reflected on his very rich scientific career of the past 50 years working and defining the field of capillary gas chromatography.

**FIGURE 1:**
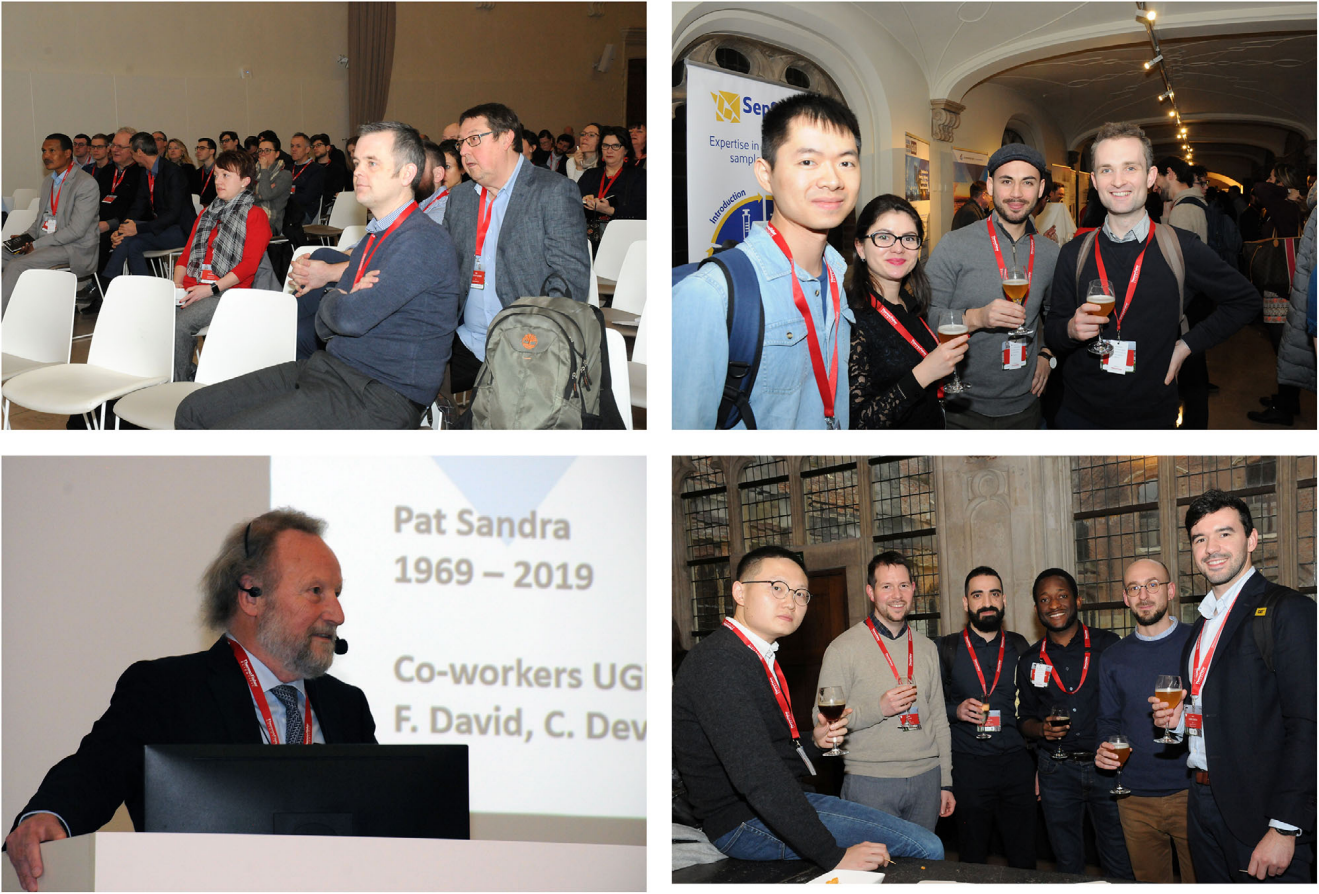
Impressions of the HTC‐16 conference held in Ghent (Belgium) from 29 till 31 January 2020. Top left: HTC lecture session; top right: beer degustation during poster session; Bottom left: Prof Pat Sandra during closing plenary lecture; bottom right: emerging young scientists

The posters presented during the conference represent a significant part of the HTC symposia. Over 75 posters were presented during different poster sessions. To promote informal discussions, one of the poster sessions was organized during an evening session and was complemented with Belgian beer tasting and selected snacks including Belgian fries. Three awards co‐sponsored by Analytical Science Advances were presented to the three best posters based on innovation, experimental work, and presentation. Therefore, poster presentations were graded in two different rounds by a group of international expert reviewers. The three poster winners were Ali Moussa (Vrije Universiteit Brussel) for numerical and experimental investigation of sample loss and dispersion occurring in sample loops used in 2D‐LC setups, Haibin Li (KU Leuven) who presented an experimental procedure for the in‐depth evaluation of band broadening phenomena in capillary and nanosize columns, and Zhanyao Hou (Ghent University) for his research on polymerization of the through‐pores in HPLC columns for enhanced SEM based assessment of packing order.

*An interview with recipient Ali Moussa (Vrije Universiteit Brussel)*

**FIGURE 2:**
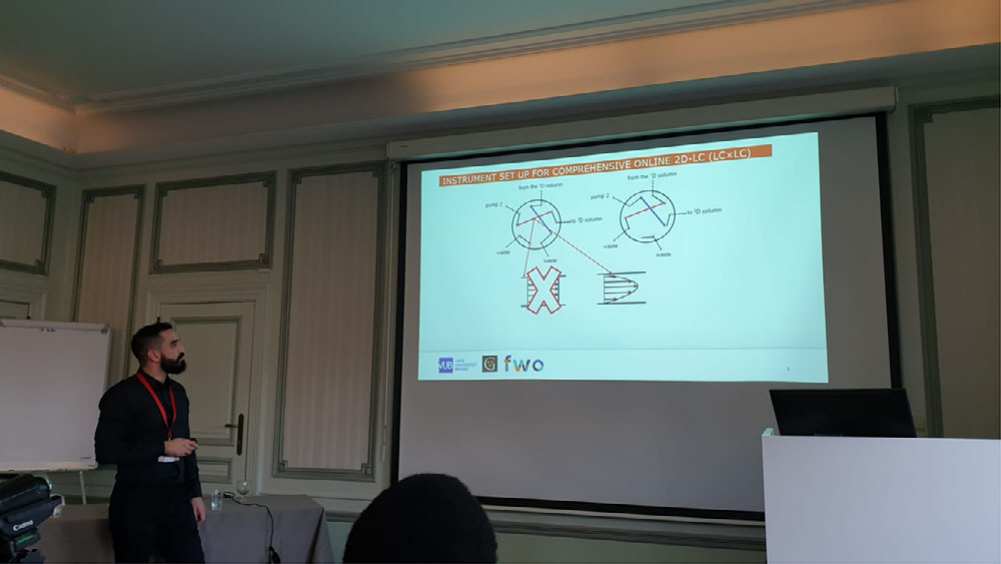
Ali Moussa presenting at the HTC‐16 conference

In 2017, I received my bachelor's degree in Chemical Engineering from the Lebanese University in my home country Lebanon. Afterward I moved to Belgium where I graduated in 2019 with a joint master's degree in Chemical Engineering from the Vrije Universiteit Brussel (VUB) and the Université Libre de Bruxelles (ULB). I did my master's thesis at the Chemical Engineering department of the VUB under the supervision of Prof Gert Desmet. The thesis research focused on studying fluid flow in a 3D‐printed microreactor using computational fluid dynamics (CFD). I continued working in the same research group as a PhD student, and my current work focuses on CFD modeling of transport phenomena in chromatographic systems.

**Would you briefly explain what your research group is studying?**

Our research group studies methods and systems used in analytical separations. Our focus is mainly on dispersion in liquid chromatography. We use both experiments and computer simulations to perform our studies. Currently I am more involved in the simulations section.

**Why did you choose a path into scientific research?**

For some kind of reason, I have always been fascinated by science and by the universe. Growing up as a kid I used to watch the stars in the clear skies and dream that 1 day I will become an astronaut. But then you grow up and you realize there are only a handful of astronauts in the world. So, it was not a very realistic dream. Then I approached science in a different way, I studied hard and I grabbed chances in life as they came along. I'm not an astronaut but a young scientist in Belgium. Not bad, I think. And who knows what the future may bring.

**What was your first experiment?**

That was a long time ago, I remember when I was a little boy in the 6th grade it was the first time we studied chemistry and the teacher gave every group of students a simple experiment to perform in class and explain. We had to mix baking soda and vinegar in a bottle to inflate a balloon fixed at the bottle opening. It sounds simple but for a 12 years old kids it was impressive!

**Who were the most influential people in your path as a scientist?**

Of course, my science teachers at school and at university played an important role to me. I had some great teachers throughout my academic years.

**What was the best advice you received from your peers?**

Best advice from peers: Grab your chance. Basically, they meant: Study hard, work hard and if you get a chance to improve your career: Don't hesitate too long but take it.

**What do you consider to be the more exciting topics in your field?**

What excites me the most in my field is doing studies where we combine both experiments and computer simulations. Being able to predict experimental results using computer simulations is impressive. It allows us to generate results faster and at a reduced cost.

**What are your views on the future of your field?**

There is a big future ahead in the field of analytical separations; the more complicated chemical and pharmaceutical products we produce, the better separation techniques we need to purify these products. Also, biological samples are becoming more complex and there is a need to separate these samples to the best extent possible. I think that computer simulations will be used more in the future to design methods and techniques used in analytical separations.

**What is your favorite pastime outside of science?**

My favorite pastimes outside my field are travelling to learn more about others and explore new places, jogging and going to the gym to stay fit. Recently, I started picking up yoga.

**What would you do if you had 1‐year paid leave?**

I'm only 26 so I actually wouldn't like to have a year off. At this stage of my life I want to work more and keep focusing on my career. Taking a year off now feels to me like wasting a year. But do ask me again this question when I'm older.

**What nonscientist inspires you the most?**

Definitely my father. My dad had to work at an early age to support his parents and siblings, so he is a very hard‐working person. Now all these years later he still has the work ethics of a clock. He taught me to work hard and never take things for granted.

*An interview with recipient Haibin Li (KU Leuven)*

**FIGURE 3:**
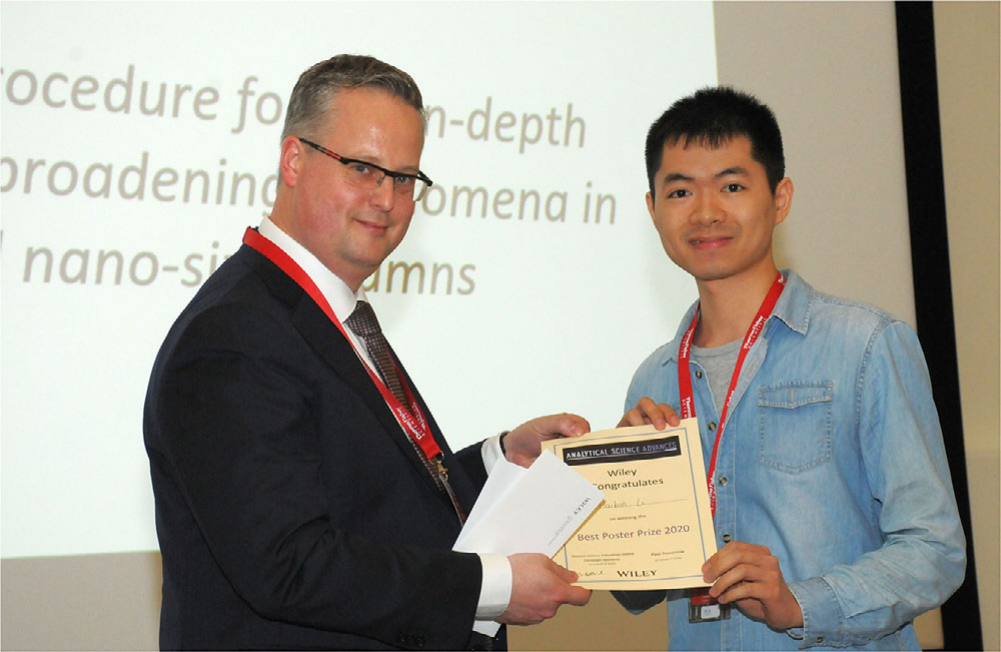
Haibin Li (right) receiving the ASA poster award

**Would you briefly explain what your research group is studying?**

Our research group led by Professor Cabooter is directed towards a deeper fundamental understanding of mass transfer phenomena in liquid chromatography, the evaluation of novel supports in chromatography, and the development of generic, automatable method development strategies for the analysis of complex samples in pharmaceutical, bio‐analytical and environmental applications.

**Why did you choose a path into scientific research?**

It is full of chances. Just like the movie lines in Forrest Gump:” Life is like a box of chocolates; you never know what you're going to get.” I didn't know what my life was going to be like when I was a child. My pharmaceutical analysis teacher (Prof Fenyun Song) during my undergraduate studies had a great impact on my life to choose a path into scientific research. Actually, I would not have become a graduate student without her. She taught us many things about the postgraduate life and the life in the lab. I think most of the undergraduates know little about the laboratory life and scientific research. Under her guidance, most of my classmates including myself participated in the postgraduate entrance examination. At the time I finished my master degree's course, it was easy to decide to do a PhD. Scientific research is great fun. Exploring the unknown world gives you a sense of accomplishment.

**What was your first experiment?**

I had some experimental courses already in high school. But all of them were designed by the teachers. My first experiment in its real sense was the determination of naringin in Juhong Huatan pills by RP‐HPLC which was the focus of my undergraduate thesis research. It was the first time that I designed the whole experiment by myself. And because the HPLC system was equipped with a manual injector rather than an autosampler, I stayed in the lab all night one day to finish the method validation. I worked in the lab for one month together with my two classmates at that time. It is highly memorable for me.

**Who were the most influential people in your path as a scientist?**

Prof Zhengjin Jiang (Jinan University) and Prof. Deirdre Cabooter (KU Leuven) had a profound influence on me. I do remember the first time I met prof. Jiang in his office. At that time, I was like a blank paper and knew nothing about scientific research. He has changed me and, in the meantime, I have learned a lot from him. He is a precise man with logic and he is my enlightenment teacher. Prof Cabooter is another wonderful mentor of mine, and my friend. She is kind, supportive, extremely dedicated and hard working. She is always encouraging me when I encounter some problems in my experiments telling me not to worry. You will not feel stressed when working with her.

**What was the best advice you received from your peers?**

“Love your work and love your life.” Do your work with your whole heart? Love your job or change it.

**What do you consider to be the more exciting topics in your field?**

Intact protein analysis and monoclonal antibody analysis. As small molecule analysis becomes easier, biological macromolecules will be the hot spot in the future. And therapeutic monoclonal antibodies are widely used in oncology nowadays.

**What are your views on the future of you field?**

Faster, miniaturized and simpler measurements in analytical science. For example, if we want to check our body, we need to make an appointment, go to the hospital, and then wait two hours or even one day to get the report. I hope that in the near future, analytical science can enter our lives. Maybe we can conduct conventional tests simply at home with a cheap apparatus rather than in the lab using an expensive, large and ponderous instrument. Just like in the scene in the famous film Big hero 6. It takes time, of course, but I think it will become reality in the future.

**What are your favorite pastimes outside of science?**

I like sports generally, especially basketball. During my high school period, I played basketball with my friends every weekend. But it's hard to get together now, because we live in different cities and have different things to do. However, we often talk about basketball and watch basketball matches on TV.

**What would you do if you had 1‐year paid leave?**

I would travel with my parents. They worked hard every day for me in the past. I think I would like to go with my parents for a year to see all of China.

**What nonscientist inspires you the most?**

Someone that I admire very much is Bruce Lee. He is a famous action actor. Bruce Lee was the iconic international superstar that popularized Chinese martial arts in the West. He has caught fire around the world. I think many people know the word “kung fu” because of Bruce Lee. No one can surpass his movies, though the movie stunts are very developed now.

*An interview with Zhanyao Hou (Ghent University)*

**FIGURE 4:**
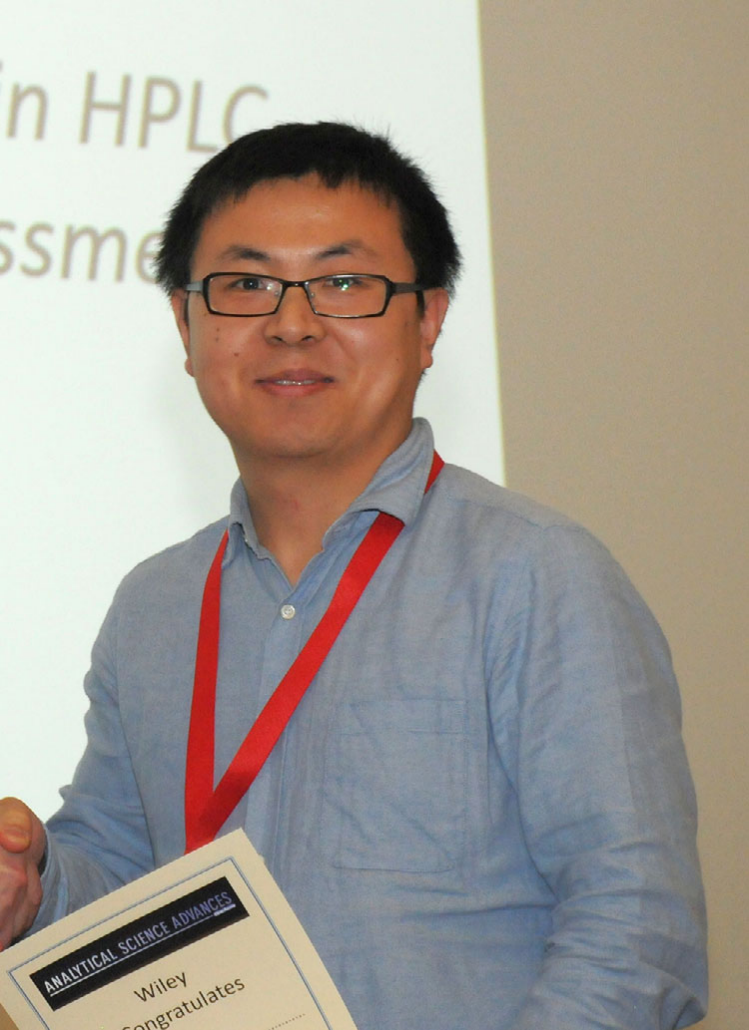
Dr Zhanyao Hou awarded with the ASA poster price

I was born in Henan, China and received my Master's degree in polymer chemistry and physics from Nankai University in 2013. My master topics mainly focused on the synthesis and physical characterization of the hybrid cluster. In February 2014, I joined in Supramolecular Chemistry Group of Ghent University to conduct PhD research on supramolecular chemistry under the supervision of Prof. Richard Hoogenboom. I received my doctoral degree in 2018 and stayed in the group as a postdoctoral researcher for 3 months. In January 2019, I started in my current position in separation science group (SSG) as a postdoctoral researcher under the advisor of Prof. Frederic Lynen. My current research topic is the investigation of the thermo‐responsive LC columns.

**Would you briefly explain what your research group is studying?**

My current research group, Separation Science Group (SSG) aims at developing novel chromatographic, electrophoretic, and mass spectrometric strategies for enhanced analysis, purification or activity assessment of organic solutes. The group specializes in novel column chemistries (e.g., HPLC, GC, CEC, SFC), sample preparation approaches, the development of multidimensional strategies for the separation of complex samples of natural and synthetic origin and in the development of predictive algorithms allowing improved selectivity tuning in chromatography. The work on high‐end applications focuses on the development of qualitative and quantitative methods, nontargeted approaches, fast UHPLC and on the implementation of hyphenated analytical platforms comprising chromatography and mass spectrometry in life sciences.

**Why did you choose a path into scientific research?**

During my Bachelor study, I started to enter the laboratory as a research assistant in the second year, and I was deeply attracted by the chemistry experiments. Although I still cannot say that I know exactly where I'm heading in terms of career. What I am fairly certain of, however, is that my future lies in science.

**What was your first experiment?**

My first experiment is the synthesis of organic phosphoric acid, which I performed by myself in the third year of my Bachelor study.

**Of all your research projects, which one was your favorite and why?**

Over the past 10 years, I have done around 7 projects in a wide range of research areas, such as organic synthesis, polymer chemistry and analytical chemistry. Most projects are purely academic research. But I hope that my research will find practical applications. My current project is to manufacture thermo‐responsive HPLC columns, which is the most close to application. My dream is that this kind of column will be commercialized in the near future.

**Who were the most influential people in your path as a scientist?**

My supervisor during my master's degree, Prof. Wei Wang, influenced me the most on my research path. His attitude and approach to research influenced me and let me know how to be a scientific researcher.

**What are your favorite pastimes outside of science?**

My spare times is mostly spent with my family. It is great to play with my kids that are 6 months and 3 years old. It's interesting and exciting to see how they discover the world around them.

**What would you do if you had 1‐year paid leave?**

If I have one‐year paid leave, I will travel around the world. In the past few years, I rarely travelled due to my busy work. I am looking forwards to this chance.

